# The iron-sulfur scaffold protein HCF101 unveils the complexity of organellar evolution in SAR, Haptista and Cryptista

**DOI:** 10.1186/s12862-021-01777-x

**Published:** 2021-03-19

**Authors:** Jan Pyrih, Vojtěch Žárský, Justin D. Fellows, Christopher Grosche, Dorota Wloga, Boris Striepen, Uwe G. Maier, Jan Tachezy

**Affiliations:** 1 Department of Parasitology, Faculty of Science, Charles University, BIOCEV, Průmyslová 595, 25250 Vestec, Czech Republic; 2 Department of Cellular Biology, University of Georgia, Athens, GA USA; 3 Laboratory for Cell Biology, Philipps University Marburg, Karl-von-Frisch-Str. 8, 35032 Marburg, Germany; 4 LOEWE Center for Synthetic Microbiology (Synmikro), Hans-Meerwein-Str. 6, 35032 Marburg, Germany; 5 Laboratory of Cytoskeleton and Cilia Biology, Nencki Institute of Experimental Biology of Polish Academy of Sciences, 3 Pasteur Street, 02-093 Warsaw, Poland; 6 Department of Pathobiology, School of Veterinary Medicine, University of Pennsylvania, 380 South University Avenue, Philadelphia, PA 19104 USA

**Keywords:** HCF101, Ind1, Iron-sulfur cluster, Mitochondrion, Plastid, Evolution

## Abstract

**Background:**

Nbp35-like proteins (Nbp35, Cfd1, HCF101, Ind1, and AbpC) are P-loop NTPases that serve as components of iron-sulfur cluster (FeS) assembly machineries. In eukaryotes, Ind1 is present in mitochondria, and its function is associated with the assembly of FeS clusters in subunits of respiratory Complex I, Nbp35 and Cfd1 are the components of the cytosolic FeS assembly (CIA) pathway, and HCF101 is involved in FeS assembly of photosystem I in plastids of plants (chHCF101). The AbpC protein operates in Bacteria and Archaea. To date, the cellular distribution of these proteins is considered to be highly conserved with only a few exceptions.

**Results:**

We searched for the genes of all members of the Nbp35-like protein family and analyzed their targeting sequences. Nbp35 and Cfd1 were predicted to reside in the cytoplasm with some exceptions of Nbp35 localization to the mitochondria; Ind1was found in the mitochondria, and HCF101 was predicted to reside in plastids (chHCF101) of all photosynthetically active eukaryotes. Surprisingly, we found a second HCF101 paralog in all members of Cryptista, Haptista, and SAR that was predicted to predominantly target mitochondria (mHCF101), whereas Ind1 appeared to be absent in these organisms. We also identified a few exceptions, as apicomplexans possess mHCF101 predicted to localize in the cytosol and Nbp35 in the mitochondria. Our predictions were experimentally confirmed in selected representatives of Apicomplexa (*Toxoplasma gondii*), Stramenopila (*Phaeodactylum tricornutum*, *Thalassiosira pseudonana*), and Ciliophora (*Tetrahymena thermophila*) by tagging proteins with a transgenic reporter. Phylogenetic analysis suggested that chHCF101 and mHCF101 evolved from a common ancestral HCF101 independently of the Nbp35/Cfd1 and Ind1 proteins. Interestingly, phylogenetic analysis supports rather a lateral gene transfer of ancestral HCF101 from bacteria than its acquisition being associated with either α-proteobacterial or cyanobacterial endosymbionts.

**Conclusion:**

Our searches for Nbp35-like proteins across eukaryotic lineages revealed that SAR, Haptista, and Cryptista possess mitochondrial HCF101. Because plastid localization of HCF101 was only known thus far, the discovery of its mitochondrial paralog explains confusion regarding the presence of HCF101 in organisms that possibly lost secondary plastids (e.g., ciliates, *Cryptosporidium*) or possess reduced nonphotosynthetic plastids (apicomplexans).

**Supplementary Information:**

The online version contains supplementary material available at 10.1186/s12862-021-01777-x.

## Background

Iron-sulfur (FeS) cluster assembly pathways are essential for all three domains of life: Bacteria, Archaea, and Eukarya. In eukaryotes, there are three main pathways, which are localized in distinct cellular compartments: mitochondria, plastids, and the cytosol. The organellar pathways were acquired through endosymbiosis of α-proteobacteria and cyanobacteria that evolved into mitochondria and plastids, respectively [1, 2]. The mitochondrial FeS cluster assembly (ISC) machinery operates in nearly all forms of mitochondria including anaerobic hydrogenosomes [3] and highly reduced mitosomes [4]. The pathway in plastids is called the sulfur utilization factor (SUF) system, which is present in primary [5] as well as secondary plastids [6–8]. The ISC machinery is functionally linked to the third system, the cytosolic FeS cluster assembly (CIA) machinery. Phylogenetic analysis suggested that the CIA pathway was present in the last eukaryotic common ancestor (LECA) and that its components are predominantly of bacterial origin [9, 10]. There are few known exceptions to the highly conserved setup of FeS assembly machineries, and all these exceptions concern protists adapted to anaerobic or microaerobic conditions with modified mitochondria [11]. Archamoebae replaced the ISC pathway with two components of a nitrogen-fixing (NIF) machinery that were acquired by lateral gene transfer (LGT) from ɛ-proteobacteria [12]. The NIF system operates in the cytosol of *Entamoeba histolytica* or in the cytosol and hydrogenosomes of *Mastigamoeba balamuthi* [13]. Similarly, the breviate *Pygsuia biforma* apparently replaced the ISC system with an archeal SUF system [14, 15]. Finally, three SUF components (SufC, SufB, and fused protein SufDSU) of bacterial origin were found in the cytosol of the oxymonad *Monocercomonoides *sp., which lost its mitochondria [11, 16].

The only proteins that are common to the CIA, ISC, and SUF pathways are P-loop nucleoside-triphosphatase (NTPases) of Mrp (MetG-related protein)/nucleotide-binding protein 35 (Nbp35) subclass with ParA domain: Nbp35/cytosolic FeS cluster deficient 1 (Cfd1), Ind1, and high chlorophyll fluorescence 101 (HCF101)(hereafter Nbp35-like proteins) [17]. In CIA, Nbp35/Cfd1 proteins serve in the initial phase of FeS assembly as a [4Fe-4S] scaffold potentially using a yet unknown component exported from mitochondria [18]. The FeS cluster is then transferred via Nar1 and the Cia1/Cia2/MMS19 targeting complex to apo-proteins. The ISC component Ind1 serves as a scaffold in later stages of FeS assembly to deliver [4Fe-4S] clusters specifically to the apo-subunits of mitochondrial respiratory Complex I, and thus, the presence of Ind1 closely matches the Complex I distribution [19]. Its necessity for Complex I maturation underlines the presence of Ind1 in hydrogenosomes, in which Complex I is reduced to only two FeS subunits [15, 20]. HCF101 was shown to transport [4Fe-4S] clusters to photosystem I subunits and heterodimeric ferredoxin-thioredoxin reductase complexes in plastids of *Arabidopsis thaliana* [21, 22].

It is believed that the cannonical distribution of FeS cluster assembly machineries and thus that of machinery-specific Nbp35-like proteins is highly conserved in eukaryotes, including protists with primary or complex plastids. The latter organelles evolved in eukaryotic hosts from eukaryotic symbionts with green (Euglenophyceae and Chlorarachniophyceae) or red (Stramenopila, Alveolata, Haptophytes, and Cryptophytes) plastids [23, 24]. These complex plastids are surrounded by three or more membranes and characterized by the presence of a periplastidal compartment, the extremely reduced cytosol of the endosymbiont [25, 26] and, in the case of cryptophytes and chlorarachniophytes, of a remnant nucleus (nucleomorph) [27]. Interestingly, the presence of nucleomorph, which is likely dependent on activities of FeS proteins, correlates with the presence of the endosymbiotic CIA, including Nbp35 that is retained in the periplastidial compartment [7] in addition to CIA in the host cytosol. This curious finding further exemplifies the conserved topology of Nbp35 and other CIA components.

A conserved distribution of Nbp35-like proteins and particularly HCF101 in plastids makes these proteins suitable candidates to study evolutionary history of eukryotes with the complex plastids that is still a matter of discussion. According to the Chromalveolate hypothesis, a single endosymbiosis with an algae of the red lineage have rise to secondary plastids in Stramenopila, Alveolata, Haptophytes, and Cryptophytes that altogether constitute the group Chromalveolata [28]. This hypothesis postulates that a single event for the establishment of a plastid was more parsimonious than a multiple plastid origin, and the absence of plastid in a few lineages (e.g. cilliates, oomycetes) was explained by the secondary plastid loss. Monophyly of Chromalveolata was supported mainly by analyses of genes that coded plastid components [29, 30] and by the presence of symbiont-derived endoplasmic-reticulum-associated protein degradation (ERAD)-like machinery (SELMA) [25]. However, increasing number of related lineages that appeared to lack plastid (e.g. katablepharids, goniomonas, centrohelids, telonemids) became inconsistent with the Chromalveolata concept [31]. Moreover, phylogenies based on non-plastidial genes revealed the close relationship of Stramenopila, Alveolata, and Rhizaria (SAR) and more recently Telonemia [32]. Haptophytes and Cryptophytes appeared together as a second Chromalveolata lineage [33], however later analyses using multi-gene phylogenies showed that cryptophytes branched within Archaeplastida whereas placement of Haptista was not conclusive [32, 34, 35]. A common origin of a chromalveolata plastid was challenged by the serial hypothesis that proposed the serial endosymbiotic transfer of the red plastid to the lineages with complex plastids, whereas aplastidial lineages either never experienced the endosymbiont presence or in a few parasitic organisms the plastid was lost [31, 36].

The localization of HCF101 has not been experimentally studied in most eukaryotic lineages. Moreover, because HCF101 is essential for photosystem I and consequently photosynthesis, it could be particularly interesting to investigate its presence and cellular localization in organisms that possess non-photosynthesizing plastids such as the apicoplast in apicomplexans. The genes for HCF101 have been noticed in several apicomplexan genomes such as *Toxoplasma gondii* and *Plasmodium falciparum*, and their possible localization in the apicoplast has been suggested [37, 38]. However, these HCF101 homologs lack the targeting signals one would expect for proteins localized to the apicoplast [37, 38]. Even more puzzling is the identification of HCF101 in the genome of *Cryptosporidium parvum,* which has lost its plastid [38]. Therefore, we decided to search for Nbp35-like genes across eukaryotic genomes and to predict their cellular localization based on their organellar targeting presequences. In selected protists, we verified the localization of Nbp35-like proteins experimentally. The most surprising result is the identification of the mitochondrial form of HCF101 in protists with complex plastids of SAR, Haptista and Cryptista lineages which explains confusion regarding the presence of HCF101 in lineages without plastids.

## Results

### Distribution of Nbp35-like proteins in eukaryotes

We searched for Nbp35, Cfd1, Ind1, and HCF101 in genomes and transcriptomes across the main eukaryotic lineages, and for each protein we predicted its putative cellular localization (Table 1, Additional file: Table S1). While Nbp35 was found ubiquitously in all lineages as reported previously [10], Cfd1 was generally present in Ophistokonta, Amoebozoa, Cryptista, Glaucophypta (Archaeplastida), Metamonada, and Discoba but absent in Chloroplastida and Rhodophyta (Archaeplastida), SAR, and Haptista. Diplomonads such as *Giardia intestinalis* and *Spironucleus salmonicida* represent the only exception within metamonads as they lack Cfd1 (Table 1) [39]. Nbp35 homologs were not identified in only four organisms, most likely due to the incompleteness of the available sequencing data (Table 1). As expected, the cytosolic localization was predicted for all Cfd1 proteins, and majority of Nbp35 proteins with a few interesting exceptions. In *Mastigamoeba balamuthi*, there are three Nbp35 paralogs, of which one was predicted to possess N-terminal hydrogenosomal targeting presequences (Table 1). Furthermore, we also predicted a mitochondrial targeting signal for Nbp35 proteins in Apicomplexa and Chromerids.

**Table 1:** Identification of Nbp35-like proteins in selected representatives of eukaryotic lineages and prediction of their cellular localization

Supergroup	Group/Species	Cytosol	Mitochondria	Chloroplast	
				Green	**Red**
**Ophistokonta**	**Metazoa**				
	*Homo sapiens*	**Nbp35, Cfd1**	**Ind1**		
	*Drosophila melanogaster*	**Nbp35, Cfd1**	**Ind1**		
	**Fungi**				
	*Saccharomyces cerevisiae*	**Nbp35, Cfd1**			
	*Yarrowia lipolytica*	Nbp35, Cfd1	**Ind1**		
**Amoebozoa**	**Lobosa**				
	*Acanthamoeba castelanii*	Nbp35, Cfd1	Ind1		
	**Conosa**				
	*Dictyostelium discoideum*	Nbp35, Cfd1	Ind1		
	*Mastigamoeba balamuthi*	Nbp35, Cfd1			
	*Entamoeba histolytica*	Nbp35, Cfd1			
**Archaeplastida**	**Viridiplantae**				
	*Arabidopsis thaliana*	**Nbp35**	**Ind1**	**chHCF101**	
	*Chlorella variabilis*	Nbp35	Ind1	chHCF101	
	*Coccomyxa subellipsoidea*	Nbp35, chHCF101	Ind1#		
	*Micromonas pusilla*	Nbp35	Ind1	chHCF101	
	*Oryza sativa*	Nbp35	Ind1	chHCF101	
	*Ostreococcus tauri*	Nbp35, chHCF101	Ind1		
	*Physcomitrella patens*	Nbp35	Ind1	chHCF101	
	*Pyramimonas parkeae*	Nbp35	Ind1*	chHCF101	
	*Selaginella moellendorffii*	Nbp35	Ind1#	chHCF101#	
	**Glaucophyta**				
	*Glaucocystis *sp.	Nbp35	Ind1		chHCF101*
	*Gloeochaete wittrockiana*	Nbp35*, Cfd1*	Ind1*		chHCF101
	**Rhodophyta**				
	*Chondrus crispus*	Nbp35	na		chHCF101
	*Cyanidioschyzon merolae*	Nbp35	Ind1		chHCF101
	*Erythrolobus madagascarensis*	Nbp35*	Ind1*		chHCF101*
	*Galdieria sulphuraria*	Nbp35	Ind1		chHCF101
	*Madagascaria erythrocladiodes*	Nbp35*	na		chHCF101
	*Porphyridium aerugineum*	Nbp35	Ind1		chHCF101*
	*Rhodosorus marinus*	Nbp35*	Ind1*		chHCF101*
	*Timspurckia oligopyrenoides*	na	Ind1		chHCF101*
**SARCH**					
** Alveolata**	**Apicomplexa**				
	*Babesia bovis*	mHCF101*	Nbp35#		
	*Cryptosporidium muris*	mHCF101	Nbp35#		
	*Toxoplasma gondii*	**mHCF101**	**Nbp35**		
	*Plasmodium falciparum*	mHCF101	Nbp35#		
	*Plasmodium yoelii*	mHCF101	Nbp35#		
	**Chromerida**				
	*Chromera vellia*	mHCF101	Nbp35#		chHCF101
	*Vitrella brassica*	mHCF101	Nbp35#		chHCF101
	*Perkinsus marinus*	Nbp35#	mHCF101#		
	**Dinoflagellata**				
	*Alexandrium monilatum*	Nbp35*	mHCF101*	chHCF101*	
	*Alexandrium tamarense*	Nbp35*	mHCF101	chHCF101	
	*Durinskia baltica*	Nbp35	na		chHCF101*
	*Dinophysis acuminata*	Nbp35*	mHCF101*		chHCF101*
	*Karenia brevis*	Nbp35*	mHCF101		chHCF101
	*Oxyrrhis marina*	Nbp35*	mHCF101*		na
	*Symbiodinium *sp.	Nbp35#	mHCF101*	chHCF101	
	**Cilliata**				
	*Tetrahymena thermophila*	**Nbp35**	**mHCF101**		
	*Oxytricha trifallax*	Nbp35	mHCF101		
	*Paramecium tetraurelia*	Nbp35	mHCF101		
	*Stylonychia *sp.	Nbp35	mHCF101		
** Rhizaria**	**Rhizaria**				
	*Ammonia *sp.	Nbp35	mHCF101*		
	*Elphidium margaritaceum*	Nbp35	mHCF101*		
	*Reticulomyxa filosa*	Nbp35	mHCF101		
	*Paulinella chromatophora*	na	na	Bacterial HCF101-like	
	**Chlorarachniophyta**				
	*Bigelowiella longifila*	Nbp35	mHCF101*		chHCF101*
	*Bigelowiella natans*	Nbp35*	mHCF101*		chHCF101
	*Lotharella globosa*	Nbp35*	mHCF101*		chHCF101*
** Stramenopila**	**Oomycota**				
	*Albugo candidagi*	Nbp35	mHCF101#		
	*Albugo laibachii*	Nbp35	mHCF101#		
	*Aphanomyces astaci*	Nbp35	mHCF101		
	*Aphanomyces invadans*	Nbp35	mHCF101		
	*Phytophthora infestans*	Nbp35	mHCF101*		
	*Phytophthora parasitica*	Nbp35	mHCF101		
	*Saprolegnia diclina*	Nbp35	mHCF101		
	**Ochrophyta**				
	*Aureococcus anophagefferens*	Nbp35	mHCF101*		chHCF101*
	*Ectocarpus siliculosus*	Nbp35#	mHCF101		chHCF101
	*Phaeodactylum tricornutum*	**Nbp35**	**mHCF101**		**chHCF101**
	*Nannochloropsis gaditana*	Nbp35	mHCF101		chHCF101
	*Schizochytrium aggregatum*	Nbp35	mHCF101		na
	*Thalassiosira oceanica*	Nbp35	mHCF101*		chHCF101
	*Thalassiosira pseudonana*	Nbp35	mHCF101		chHCF101
	*Blastocystis hominis*	**Nbp35**	mHCF101		
** Haptista**	**Haptophyta**				
	*Chrysochromulina polylepis*	Nbp35*	mHCF101*		chHCF101*
	*Emiliania huxleyi*	Nbp35	mHCF101		chHCF101
	*Exanthemachrysis gayraliae*	na	mHCF101*		chHCF101*
	*Gephyrocapsa oceanica*	Nbp35	mHCF101*		chHCF101
	*Isochrysis galbana*	Nbp35	mHCF101		chHCF101*
	*Pleurochrysis carterae*	Nbp35	mHCF101		chHCF101*
	*Prymnesium parvum*	Nbp35*	mHCF101*		chHCF101*
	**Centrohelida**				
	*Raineriophrys erinaceoides*	Nbp35*	mHCF101*		
** Cryptista**	**Cryptophyta**				
	*Chroomonas mesostigmatica*	Nbp35*, Cfd1*	mHCF101*		chHCF101*
	*Cryptomonas curvata*	na	mHCF101		na
	*Cryptomonas paramecium*	Nbp35*, Cfd1*	mHCF101		na
	*Geminigera cryophila*	Nbp35*, Cfd1	mHCF101		chHCF101*
	*Guillardia theta*	Nbp35/Cfd1	mHCF101*		chHCF101#
	*Hanusia phi*	Nbp35*, Cfd1*	mHCF101		na
	*Hemiselmis rufescens*	Nbp35, Cfd1*	mHCF101*		chHCF101#
	*Proteomonas sulcata*	Nbp35*, Cfd1	mHCF101*		na
	*Rhodomonas *sp.	Nbp35, Cfd1*	na		chHCF101*
	**Katablepharida**				
	*Roombia truncata*	Nbp35*, Cfd1*	mHCF101*		
	**Goniomonas**				
	*Goniomonas avonlea*	Nbp35*, Cfd1*	mHCF101		
	*Goniomonas pacifica*	Nbp35, Cfd1*	mHCF101*		
** Excavata**	**Euglenozoa**				
	*Euglena gracilis*	Nbp35, na	na	chHCF101*	
	*Eutreptiella gymnastica*	Nbp35, Cfd1	Ind1	chHCF101	
	*Trypanosoma brucei*	**Nbp35, Cfd1**	**Ind1**		
	*Leishmania major*	Nbp35, Cfd1	Ind1		
	**Metamonada**				
	*Trichomonas vaginalis*	Nbp35, Cfd1	Ind1		
	*Giardia intestinalis*	**Nbp35**	**Nbp35**		
	**Heterolobosea**				
	*Naegleria gruberi*	Nbp35, Cfd1	Ind1		

Proteins with experimentally verified localization are in bold. Underlined taxons indicate available genome sequence, transcriptomes were available for other taxons

*Incomplete sequence of the gene, cellular localization is predicted based on the phylogenetic analysis (Fig. 5)

#Prediction with low confidence; na, gene was not identified in available transcriptome

Ind1 was predicted to be present in the mitochondria of Ophistokonta, Amoebozoa, Archaeplastida, and Excavata groups except for organisms that lack Complex I (Table 1). Interestingly, we did not identify Ind1 in any organism with complex plastids. While this is not surprising for apicomplexans that lack Complex I such as *Toxoplasma gondii* and *Plasmodium falciparum* and evolutionarily related chromerids *Chromera vellia* and *Vitrella brassica*, Ind1 was also absent in all other lineages of the SAR, Haptista and Cryptista groups, despite the presence of genes for the FeS subunits of Complex I in these organisms [19].

**Fig. 1:**
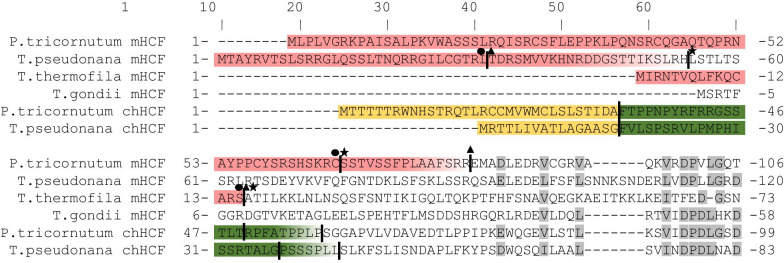
Predictions of N-terminal targeting sequences for chloroplast (chHCF101) and mitochondrial versions of HCF101 (mHCF101) in selected alveolates and stramenopiles. Red color highlights the mitochondrial leader sequence with cleavage sites predicted with Mitofates (star), TargetP 2 (circle), and PSORT II (triangle). Yellow color indicates a signal peptide, which is cleaved right before the phenylalanine residue, a typical feature for signal peptide cleavage in diatoms. This cleavage site was predicted with high support by the TargetP, Signal P, and HECTAR algorithms. Green represents a predicted transit peptide with two possible cleavage sites for stromal processing peptidase, manually predicted based on previous studies [92]. Gray color highlights conserved residues

Finally, we searched for genes encoding the HCF101 protein. This protein could be easily distinguished from other Nbp35-like proteins based on the presence of two extra domains, an N-terminal FeS cluster assembly P domain (FSCA, previously domain of unknown function DUF59) and a C-terminal DUF971 [22]. Surprisingly, the distribution of HCF101 was limited not only to lineages harboring primary plastids (Viridiplantae, Rhodophyta, Glaucophyta) or complex plastids of red (SAR, Cryptophyta, Haptophyta) or green (chlororachniophytes, euglenids and some dinoflagellates) origin, but the gene was also present in the remaining nonphotosynthetic members of SAR, Haptista, and Cryptista. Nonphotosythetic SAR members included four apicomplexans with nonphotosynthetic apicoplast, and aplastidial species *Cryptosporidium muris*, dinoflagellate *Oxyrrhis marina* that secondarily lost plastid, four species of ciliates, three rhizarians, seven oomycetes and *Blastocystis hominis*, three members of Cryptista, and a centroheliozoan of Haptista that are all without plastids (Table 1). Every photosynthetically active eukaryote possesses a gene that encodes HCF101 with either an N-terminal primary plastid targeting signal (Archaeplastida) or a bipartite signal, which targets the protein to complex plastids (chHCF101) (Fig. 1). Strikingly, in all members of SAR, Haptista, and Cryptista (formerly referred to as Chromalveolata), we found a second HCF101 paralog, with predicted mitochondrial localization (mHCF101). The only unexpected variation of this cellular localization was found in Alveolata. In apicomplexans that harbor a nonphotosynthetic apicoplast, HCF101 was predicted to reside in the cytosol, while Nbp35 possesses an N-terminal extension, which may target the protein to the mitochondria. The same cytosolic distribution of HCF101 and possibly mitochondrial Nbp35 we predicted also for evolutionarily related Chromerids that possess photosynthetic plastids, and therefore also chHCF101. In other Alveolates, such as *Perkinsus marinus,* that possesses cryptic nonphotosynthetic plastids and in ciliates that lack plastids, we predicted standard cytosolic localization for Nbp35 and mitochondrial localization of putative mHCF101, whereas chHCF101 is absent. The distribution of Nbp35, mHCF101, and chHCF101 in dinoflagellates is likely similar to that in Stramenopila and Rhizaria; however, predictions of protein localization in some dinoflagellates had low confidence.

### Experimental localization of selected Nbp35-like proteins

**Fig. 2:**
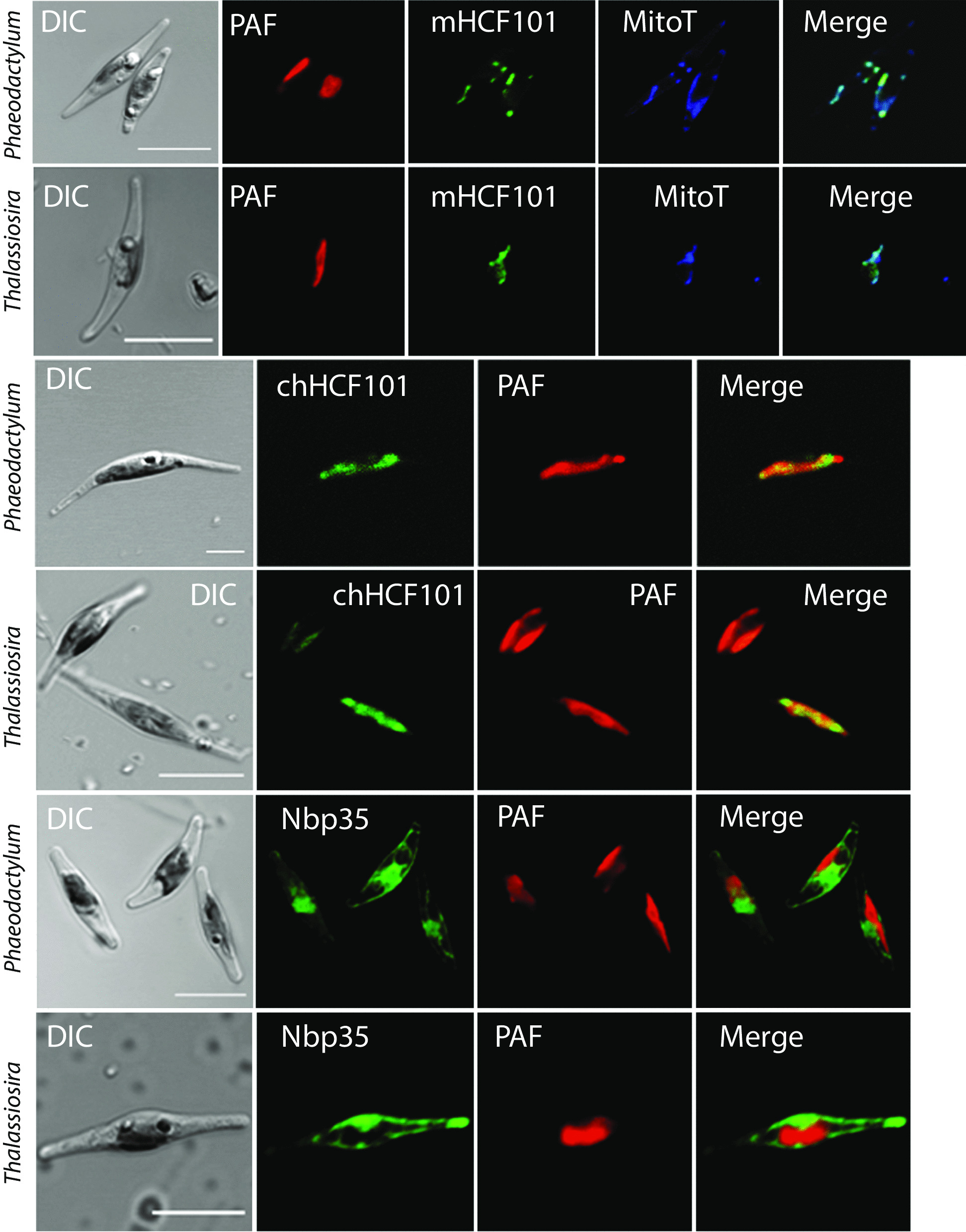
Localization of Nbp35-like proteins in diatoms. Genes for chHCF101, mHCF101, and Nbp35 from *P. tricornutum* and *T. pseudonana* were expressed in *P. tricornutum* with C-terminal e-GFP tag (green). PAF, plastid autofluorescence (red); MitoT, MitoTracker Orange (blue); DIC, differential interference contrast. Scale bar 10 µm

**Fig. 3:**
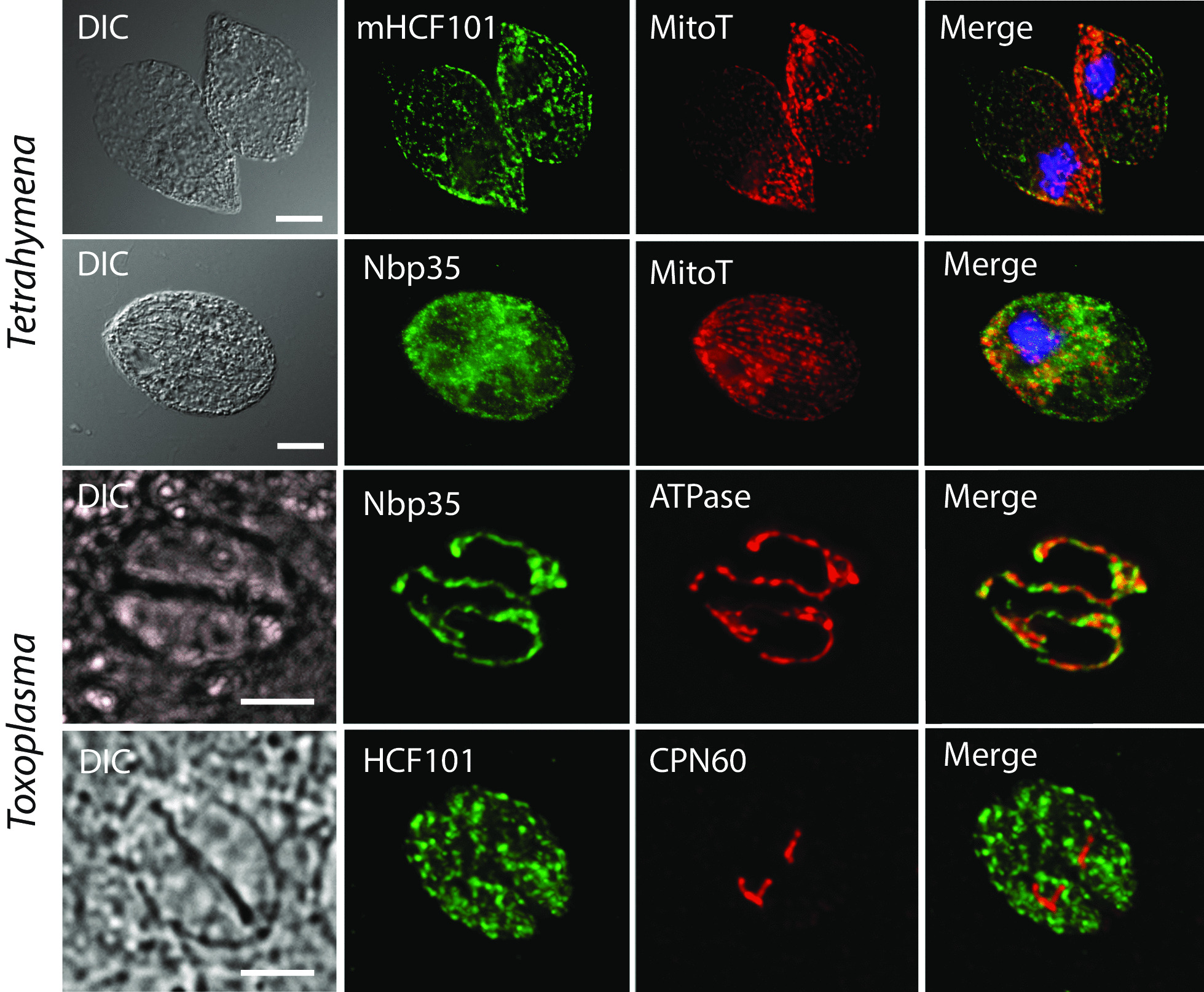
Localization of Nbp35 and mHCF101 in *T. thermophila* and *T. gondii.* Nbp35 and mHCF101 were expressed under the control of their respective native promoter with a C-terminal HA tag (green). Specific polyclonal antibodies against F1-ATPase (red), and HSP60 (red) were used as mitochondrial and apicoplastidal markers, respectively. MitoT, Mitotracker Red, (red); DIC, differential interference contrast. Scale bar 10 µm (*T. thermophila*) and 5 µm (*T. gondii*)

The identification of mHCF101 and the unexpected localization predicted for Nbp35 in apicomplexans prompted us to select three protists that are amenable for cell transformation and investigate the localization of Nbp35-like proteins using protein tagging. First, we tested genes from the diatoms *Phaeodactylum tricornutum* and *Thalassiosira pseudonana* that possess secondary plastids. *P. tricornutum* cells were transformed to express homologous eGFP-tagged mHCF101, chHCF101, and Nbp35 as well as heterologous genes from *T. pseudonana*. Fluorescence microscopy revealed that both mHCF101 proteins labeled structures that corresponded to mitochondria as indicated by colabeling with MitoTracker. These structures were clearly distinct from plastids, in which we observed labeling with chHCF101 (Fig. 2). As expected, Nbp35 labeling corresponded to the cytosol. Next, we tested localization of putative mHCF101 and Nbp35 in the ciliate *Tetrahymena thermophila,* which lacks plastids. HA-tagged mHCF101 appeared in numerous round mitochondria organized in longitudinal arrays that were again also labeled with MitoTracker (Fig. 3). Nbp35 appeared as a diffuse signal within the cell corresponding to the cytosol. Finally, we expressed HCF101 and Nbp35 in *T. gondii* (Fig. 3). This organism lacks mitochondrial Ind1 and possesses a reduced nonphotosynthetic plastid, the apicoplast. Nbp35 clearly colocalized in tubular structures with the mitochondrial marker F1-ATPase. Putative HCF101 appeared within the cell as a cytosolic protein. No localization of HCF101 to the apicoplast was observed using the antibody against plastidial chaperonin Cpn60. These experimental data confirmed the predicted localization of mHCF101 in diatoms and the ciliate and mitochondrial localization of Nbp35 in *Toxoplasma.*

### Phylogeny of HCF101

**Fig. 4:**
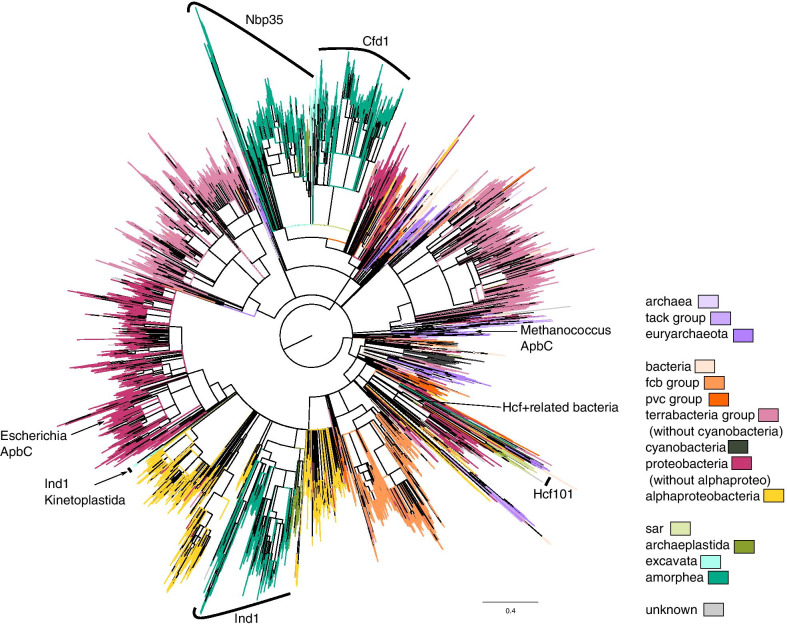
A phylogenetic tree of the Nbp35-like proteins (the ParA family). The tree was built using FastTree, and nodes with support below 0.9 were collapsed (see materials and methods). The leaves are color coded to indicate taxonomy (8840 sequences), with annotations of selected sequences. The scale bar represents the estimated number of amino acid substitutions per site

To learn about the evolutionary history of chHCF101 and mHCF101 and to obtain further support for predictions of their cellular localization, we performed phylogenetic analysis. In the first step, we were interested in the relationship between HCF101 and other members of the Nbp35-like protein family. We analyzed a large dataset of 8440 amino acid sequences including mHCF101, chHCF101, Nbp35, Cfd1, and Ind1 as well as prokaryotic homologs of alternative pyrimidine biosynthetic proteins C (ApbC) with the ParA domain. We expected that chHCF101 originated from a cyanobacterial endosymbiont that evolved to a plastid, similar to Ind1, which was acquired with the α-proteobacterial ancestor of mitochondria [9]. However, chHCF101 and mHCF101 formed a common clade with various lineages of bacteria that appeared at the base of the HCF101 subtree, including proteobacteria, the Planctomycetes, Verrucomicrobia, and Chlamydiae (PVC) group, and *Bacteroidetes*. There is no obvious support for the cyanobacterial ancestry of HCF101 and thus for endosymbiotic gene transfer (EGT), although the overall resolution of the tree is low. As expected, Ind1 formed a clade with the majority of eukaryotic sequences and α-proteobacteria at a basal position that is consistent with EGT origin of the protein (Fig. 4). Interestingly, Ind1 of kinetoplastids appeared at a separate position in the Ind1/α-proteobacterial subtree than the rest of the eukaryotic sequences, suggesting its specific phylogenetic history. It is possible that hand in hand with the presence of an atypical Complex I in kinetoplastids, Ind1 protein also underwent dramatic evolutional reshaping [40]. Finally, Nbp35/Cfd1 clustered together with various eubacterial and archaebacterial sequences as observed previously [9, 10].

In support of Nbp35-like protein clustering, we also performed prediction of protein domains with a focus on the presence of the HCF101 marker domains FSCA and DUF951 to obtain more information to estimate a possible HCF101 origin (Additional file 2: Table S2). This analysis showed that the majority of bacterial sequences have a FSCA-ParA structure (3420), or contain the ParA domain only (2762), and there are also various other domain combinations. Of note, 18 sequences obtained from proteobacteria, PVC group members, and *Bacteroidetes* clustered with eukaryotic HCF101 and shared the characteristic FSCA-ParA-DUF951 domains structure of HCF101.

**Fig. 5:**
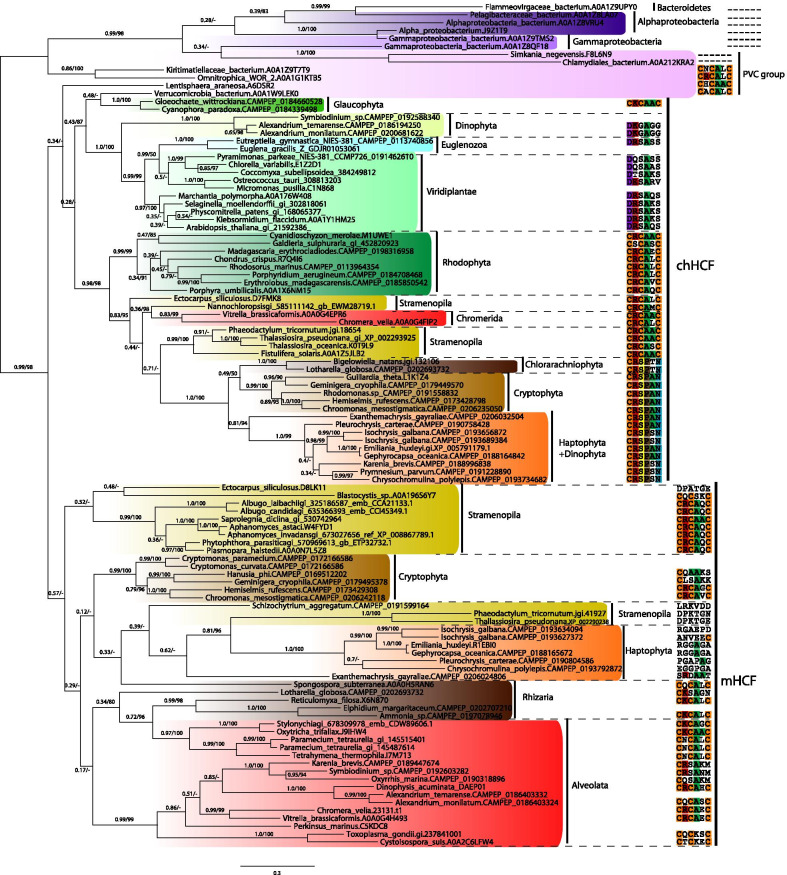
Phylogeny of HCF101 proteins. The tree was built using the PhyloBayes CAT-Poisson mixture model. Numbers at nodes of the tree indicate statistical support in the form of posterior probability of the PhyloBayes analysis and an ultrafast bootstrap of the IQ-Tree analysis (see materials and methods). The scale bar represents the estimated number of amino acid substitutions per site. The conserved cysteine motif in the DUF971 domain is displayed for each protein sequence when available

In the second step, we focused on more detailed phylogenetic analysis of chHCF101 and mHCF101 (Fig. 5). The phylogenetic tree revealed that chHCF101 and mHCF101 are paralogs that evolved from a common HCF101 ancestor, possibly by duplication events. ChHCF101 and mHCF101 formed two monophyletic groups albeit with low support (posterior probability of the PhyloBayes analysis 0.34, and 0.57 for chHCF101, and mHCF101, respectively). The chHCF101 tree is by-and-large consistent with the current concept of eukaryotic phylogeny. There are three well-supported clades of Archaeplastida with primary plastids for Viridiplantae, Glaucophyta, and Rhodophyta together with protists that harbor corresponding secondary plastids (Fig. 5). Thus, chHCF101 in Viridiplantae clusters together with Euglenozoa that possesses secondary plastids of green origin. ChHCF101 of Rhodophyta is at the base of Stramenopila, Chromerida, Cryptophytes, and Haptophytes, which contain Rhodophyta-derived red secondary plastids. However, there are some exceptions. Some dinoflagellates such *Alexandrium* and *Symbiodinium* that have secondary plastids of red origin yet seem to possess chHCF101 related to the green plastid lineage. Conversely, although chlorarachniophytes acquired secondary plastids of green ancestry, their chHCF101 clustered within orthologs of red plastids. Interestingly, this single gene phylogeny supports the close relationship between plastids of Haptophytes and Cryptophytes. Branching of the main groups was further supported by a comparison of six conserved amino acid residues (amino acid residues 461–466 according to *Arabidopsis thaliana*) in the DUF971 domain. The common motive for Viridiplantae, Euglenozoa and Dinophyta was D[K,R,Q,T][G,S]Ax[G,S], chHCF101 of Glaucophyta, Rhodophyta, Stramenophila, and Chromerida possess the highly conserved motif C[R,S]CAxC, and Chlorarachniophyta, Cryptophytes and Haptophytes possess CRSP[A,T,S]N. Because cysteine residues are crucial for the biogenesis and transfer of FeS clusters, observed differences in DUF971 domain motives may indicate important differences in their function among analyzed eukaryotic lineages.

The observed branching order of mHCF101 is poorly supported (Fig. 5); nevertheless, separation of the chHCF101 and mHCF101 groups provides a tool for our prediction of cell localization, as several sequences included in Table 1 were incomplete and thus preclude confident predictions based on the identification of N-terminal targeting motifs. For example, in dinoflagellates, we found complete sequences of two HCF101 paralogs only for *A. tamarense.* Sequences of all other dinoflagellates were incomplete; however, phylogenetic analysis clearly separated group mHCF101 including *A. tamarense* HCF101 with mitochondrial targeting presequence and formed a subtree of dinoflagellates with high statistical support. The other HCF101 dinoflagellate paralogs appeared within the chloroplast group. We were particularly interested in the origin of HCF101 of apicomplexans that lack N-terminal targeting sequences, and in *T. gondii,* we demonstrated its cytosolic localization. HCF101 proteins of *T. gondii* and other related apicomplexans including *Cystoisospora suis* clearly appeared within the mHCF101 group, at the base of a well-supported subtree of apicomplexans, chromerids, and dinoflagellates. Therefore, apicomplexan HCF101s seem not to be derived from plastids (apicoplast), contrary to previous assumptions [37, 38]. Another interesting question was the origin of HCF101 in ciliates which lack plastids. Phylogeny of HCF101 showed that HCF101 in ciliates is not related to chHCF101 but clustered within mHCF101s.

Several members of the PVC group such as *Kiritimatiellaceae bacterium* and *Verrucomicrobia bacterium* are at the base of the HCF101tree. Although this tree is poorly resolved, it is noteworthy that the bacterial conserved motif of DUF971 C[A,R,N,H]CA[A,L]C (brackets indicate an alternative) is similar to the motifs in chHCF101 as well as most mHCF101 (Fig. 5). Interestingly, the verrucomicrobial HCF101 clustered with the orthologs of the glaucophyta group, which is considered to possess the most primitive plastid. Thus, based on the phylogeny analysis, the presence of bacterial HCF101-like proteins with specific domain structures, and the conserved DUF971 motif, the subset of PVC group members represents the best candidates for the origin of eukaryotic HCF101.

## Discussion

In this work, we screened Nbp35-like homologs across eukaryotes and predicted their cellular localization. This analysis discovered the existence of a mitochondrial HCF101 homolog that is common to all tested members of SAR, Haptophytes, and Cryptophytes. Localization of mHCF101 was predicted based on the identification of N-terminal mitochondrial targeting sequences and supported by a phylogenetic analysis that separated mHCF101 from the chHCF101 paralog. Moreover, mitochondrial localization of mHCF101 was experimentally verified for mHCF101 encoded in the genomes of two diatoms (*T. pseudonana*, *P. tricornutum*) and the ciliate *T. thermophila*. Curiously, but consistently with the in silico predictions, we found mHCF101 in the cytosol of *T. gondii*, while Nbp35 was localized to the mitochondrion. Evolutionary analysis of HCF101 proteins and their specific distribution suggested that HCF101 was gained potentially via LGT from bacteria of the PVC lineage either by a common ancestor of Archaeplastida to serve in the chloroplast (plastid-first hypothesis) or by a common ancestor of Archeaplastida SAR, Haptista and Cryptista to serve first in mitochondria.

The presence of mHCF101 is coincident with the absence of Ind1, which is involved in the maturation of Complex I FeS subunits. This specific distribution suggests that mHCF101 may act as a functional homolog of Ind1. Both proteins share conserved nucleotide-binding domain characteristics of the Mrp/Nbp35 subclass of ParA P-loop NTPases [17], which includes the conserved CxxC motif (x signifies any amino acid). This motif is essential to bind the transient [4Fe-4S] cluster that is transferred to the target FeS proteins [22, 41]. It is evident that chHCF101 in chloroplasts and Ind1 in mitochondria transfers labile FeS clusters to different targets. However, both proteins are able to deliver the labile cluster to the *S. cerevisiae* model [4Fe-4S] acceptor protein, isopropyl malate isomerase, in vitro [22, 41]. Thus, the function of HCF101 proteins and Ind1 might be interchangeable. The major difference between HCF101 and Ind1 is the presence of N- and C-terminal domains in the former protein. The FSCA domain is present at the N-terminus of HCF101 (just after the N-terminal targeting sequence) and in a few other eukaryotic proteins involved in FeS assembly such as Cia2 of CIA machinery [42] and asymmetric leaves1/2 enhancer7 (AE7), which is a Cia2 homolog in *A. thaliana* [43]. The FSCA domain in combination with ParA was identified in a large number of bacterial and some archeal FeS cluster carrier proteins (this work). Importantly, in *Staphylococcus aureus*, the FSCA domain is composed solely of the SufT subunit of SUF machinery and acts as an auxiliary FeS cluster maturation factor [44]. Therefore, the fusion of FSCA and Nbp35-like protein might be beneficial for more efficient transfer of FeS centers to target proteins. The function of C-terminal DUF971 of HCF101 is currently elusive. However, we noticed that DUF971 present at the C-termini of most chHCF101 and mHCF101 proteins contains a highly conserved CxCxxC motif that may have the capacity to bind divalent metals [45]. Further studies are required to clarify a function of mHCF101 and DUF971 in particular.

**Fig. 6:**
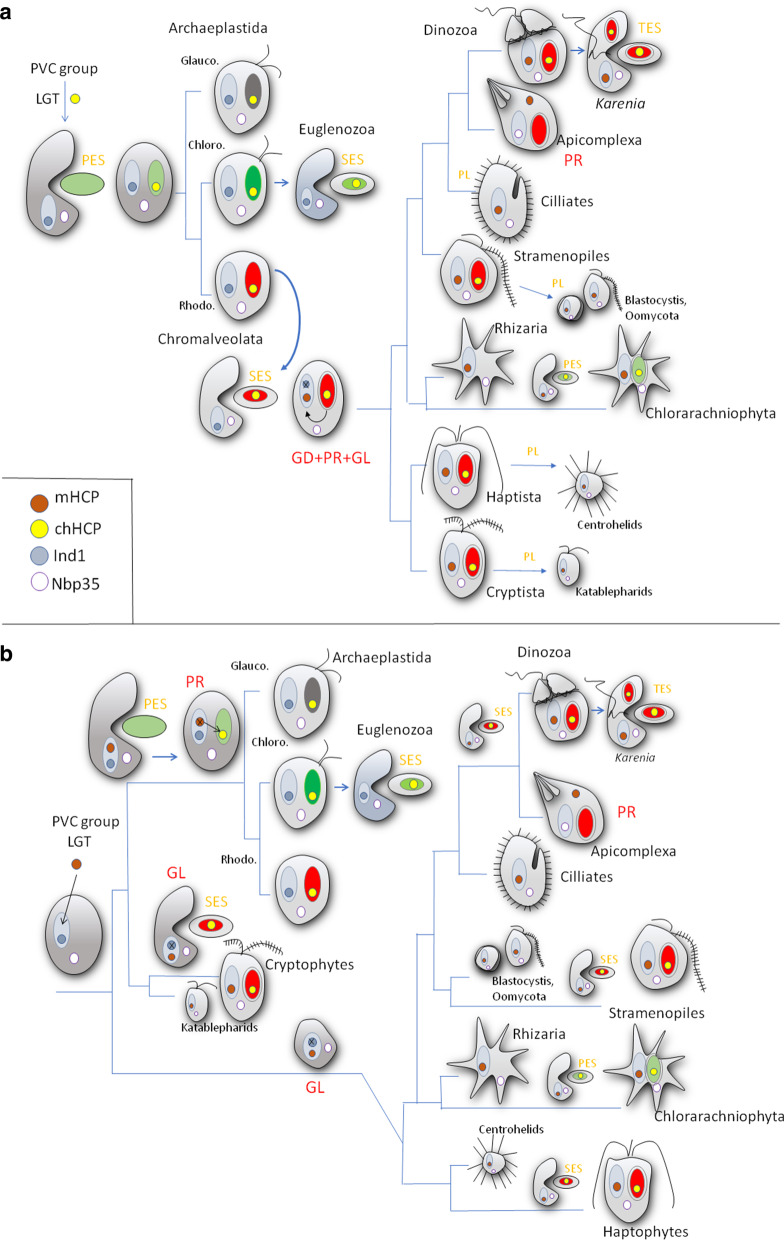
Scheme of HCF101 evolution. A. HCF101 distribution explained via the Chromalveolate hypothesis. Upon the acquisition of HCF101-like protein via LGT from bacteria to the ancestor of Archeplastida, chHCF101 was established in the plastid. Euglenozoa gained HCF101 via secondary endosymbiosis with a donor containing green plastid. A common ancestor of Chromalveolata gained red plastid via secondary endosymbiosis, the gene for chHCF101 was duplicated and one copy was targeted to mitochondria (mHCF101), where it replaced the Ind1 gene. mHCF101 is common to all chromalveolates, while several lineages lost chHCF101 together with loss of the secondary plastid (*Cryptosporidium*, Ciliates, Oomycota, centrohelids, catablepharids). B. HCF101 distribution explained via ‘multiple secondary endosymbiosis’. Mitochondrially localized HCF101 together with Ind1 was present in a common ancestor of Archaeplastida, SAR, Cryptista, and Haptista. Then, in (i) Cryptista and in (ii) a common ancestor of Haptista and SAR, the Ind1 gene was lost, whereas the Archaeplastida gene for the Ind1 protein remained in the mitochondria, and mHCF101 was retargeted to the plastid. Then, chHCF101 was introduced to Cryptophytes, Haptophytes, and certain SAR groups via multiple secondary endosymbiosis. chHCF101 is absent in lineages that did not experience secondary endosymbiosis. The schematic tree is based on previous phylogenetic studies [29, 31, 32, 36, 47, 53, 99]. PR, protein retargeting; GL, gene loss; GD, gene duplication; PL, plastid loss; PES, primary endosymbiosis; SES, secondary endosymbiosis; TES, tertiary endosymbiosis

The evolutionary journey taken by mHCF101 to arrive in the mitochondria of SAR, Haptista, and Cryptista is a puzzle, but multiple evolutionary scenarios could potentially explain the origin of this gene. Our phylogenetic and domain analyses of HCF101 proteins together with their distribution in eukaryotes suggested that ancestral HCF101 was not acquired via EGT from cyanobacteria that possess simple ParA domain-containing proteins without FCSA and DUF971. Rather, it was gained via LGT from bacteria of the PVC lineage that possessed an HCF101-like protein of the FSCA-ParA-DUF971 domain structure and cluster with chHCF101 of glaucophytes. The key question is whether HCF101 was first targeted to chloroplasts or to mitochondria. Considering the chloroplast-first scenario (Fig. 6a), we can hypothesize that HCF101 was acquired by a common ancestor of Archaeplastida, targeted to chloroplasts, and evolved independently in glaucophytes, green algae/land plants, and red algae. Then, HCF101 was transferred via secondary endosymbiosis of green plastids to Euglenophyceae and by transfer of red plastids to a putative common ancestor of SAR, Haptista and Cryptista. In this hypothetical ancestor, the HCF101 was duplicated, and one of the paralogs was targeted to mitochondria (mHCF101), where it functionally replaced Ind1. Alternatively (mitochondria-first), we can hypothesize that HCF101 was first present in the mitochondria of a common ancestor of Archeplastida, SAR, Haptista and Cryptista [46, 47] and functioned in parallel with Ind1 (Fig. 6b). HCF101 in Archaeplastida was then retargeted from mitochondria to the plastid (chHCF101), while Ind1 was lost at least twice independently in a common ancestor of Cryptophytes and of Haptophytes plus SAR.

The proposed plastid-first scenario for HCF101 evolution is consistent with the Chromalveolata hypothesis that is based on the idea that all lineages with a red secondary plastid are monophyletic [48]. In support of this hypothesis, it has been proposed that all members of chromalveolates share SELMA to target proteins into secondary plastids via the endoplasmic reticulum [25, 49]. Furthermore, remnant plastids of some seemingly aplastidal-like members of chromalveolates such as *Perkinsus marinus* were discovered [50]. In ciliates that lack plastid, several proteins of algal origin were previously identified including a MinD-like hypothetical protein in *T. thermophila* [51]. In our analysis, we identify this protein as mHCF101, and its mitochondrial localization was experimentally confirmed in *Tetrahymena*. Thus, in addition to SELMA, the presence of mHCF101 in mitochondria together with the absence of Ind1 is another feature that is common to chromalveolates.

However, an increasing number of phylogenetic studies favor multiple secondary (or serial) endosymbioses in these lineages [36, 46, 52]. They refute the chromalveolate hypothesis by placing Cryptophytes within Archeplastida and through the discovery of novel groups such as katablepharids (Cryptista) [53] and centrohelids (Haptista), in which so far no evolutionary traces of plastids have been found. Thus, their lack of plastids could reflect the primary absence of plastids rather than secondary loss [46]. Interestingly, even these lineages contain mHCF101 instead of Ind1, supporting the idea of multiple independent losses of Ind1.

Tertiary endosymbiosis is another facet that complicates tracing HCF101 evolution, particularly in dinoflagellates. Our phylogenetic analyses revealed that chHCF101 of *Karenia* clustered within the Haptophytes subtree. This is fully consistent with previous inferences that *Karenia* and related genera of dinoflagellates with the fucoxanthin-containing plastids [54, 55] lost the ancestral secondary plastid, which was replaced by a new plastid from Haptophytes via tertiary endosymbiosis [56–59]. Interestingly, another group of dinoflagellates including *Alexandrium* and *Symbiodinium* with peridin-containing plastids of red origin appeared at the base of Viridiplantae in the chHCF101 subtree, which may suggest experience with a green plastid before acquiring the red plastid, as suggested in several studies [52, 59–61]. In contrast to chHCF101 phylogenies, the monophyletic origin of mHCF101 was observed for both groups of dinoflagellates regardless of the multiendosymbiotic events, which clearly reflected different evolutionary histories for mHCF101 and chHCF101.

Another example of the complex evolution of chHCF101 is found in Chlorarachniophytes, which possess a complex plastid of green origin [28]. Perplexingly, the phylogeny of chHCF101 suggested a red origin for this protein, which clustered with Haptophytes and Cryptophytes and shared the unique CRSP[T,A,S]N motif of DUF971. However, this finding may not be so surprising. Previous analyses of the chlorarachniophyte *Bigellowiella natans* classified several genes of likely algal origin to be potentially acquired from the red lineage [62]. These ‘red’ genes are rather puzzling, but might have originated from cryptic endosymbioses involving red algae prior to the more recent acquisition of a green lineage endosymbiont [63, 64].

Based on our and previous analyses [10], Nbp35 seems to be the only essential FeS cluster assembling P-loop ATPase present in all eukaryotic cells. Typically Nbp35 is a cytosolic member of the CIA machinery; however, there are multiple examples of mitochondrial localization. In this work, we demonstrated targeting of a single Nbp35 to the *T. gondii* mitochondrion, and we similarly predicted mitochondrial localization for other apicomplexans and chromerids based on their targeting signals. Three Nbp35 genes were observed in the unrelated free-living archamoebae *M. balamuthi,* from which a single Nbp35 paralog possesses the mitochondrial/hydrogenosomal targeting sequence, and its hydrogenosomal localization was supported by previous proteomic data [65]. Dual mitosomal/cytoplasmic localization of two out of three Nbp35 paralogs was observed in metamonad *G. intestinalis* [39]. A common property shared by these organisms with mitochondrion-associated Nbp35 is that they lack Complex I and Ind1. It is tempting to speculate that mitochondrial Nbp35 replaces Ind1 and serves in the delivery of [4Fe-4S] clusters to proteins other than Complex I subunits. However, Ind1 is highly specific for Complex I, and its involvement in the maturation of other FeS proteins was not observed [19, 41].

## Conclusions

The searches for Nbp35-like proteins across eukaryotic lineages revealed mitochondrial HCF-101 homologs that are present exclusively in SAR, Haptista, and Cryptista. Thus, the presence of mHCF101 and lack of Ind1 are the first nonplastidial common features of these lineages formerly grouped under chromalveolates. Phylogeny of the HCF101 protein suggested that both mHCF101 and chHCF101 are paralogs and that an ancestral HCF101 more likely was gained by LGT from bacteria than via EGT.

## Methods

### *Toxoplasma gondii* cultivation, genetic manipulation, and microscopy

Tachyzoites of *T. gondii* derived from strain RH were cultivated and genetically manipulated as described previously [66]. HCF101 (TGME49_318590) and Nbp35 (TGME49_280730) coding sequences were amplified from *T. gondii* complementary DNA (cDNA) and cloned in frame with a triple hemagglutinin (HA) epitope tag at the 3′ end into plasmid pDt7s4HA. The constructs were transiently transfected into the *T. gondii* Δku80/TATi strain [67] using a BTX ECM 630 electroporator (Harward Apparatus). Confluent human foreskin fibroblasts (HFF) were infected with transfected parasites and fixed after 24 h of infection with 4% formaldehyde and permeabilized with 0.2% Triton X-100. Immunofluorescence microscopy was performed using the primary antibodies anti-HA (Roche), mouse anti- *T. gondii* mitochondrial F1-adenosinetriphosphatase (ATPase) [68], and rabbit anti-apicoplast Cpn60 [69]. Secondary antibodies used were goat anti-rat Alexa Fluor 488, goat anti-mouse Alexa Fluor 546, and goat anti-rabbit Alexa Fluor 546. Images were obtained on an Applied Precision Delta Vision microscope and were deconvolved and adjusted using Softworx software (GE Healthcare).

### *Tetrahymena thermophila* cultivation, genetic manipulation, and microscopy

*Tetrahymena thermophila* CU428 strain was cultivated axenically in SPP medium (1% proteose-peptone, 0.2% glucose, 0.1% yeast extract, and 0.003% ferric-sodium: ethylenediamine tetraacetic acid (EDTA) supplied with an antibiotic–antimycotic mix (Invitrogen, Carlsbad, CA) [70]. The insertion of transgenes into the *T. thermophila* macronucleus was performed as described previously [71]. Genes coding for Nbp35 (XP_001033404, TTHERM 0312220) and mHCF101 (XP_001007903, TTHERM 00538790) were amplified from genomic DNA and inserted into the pFAP44-3HA vector [72], which allows the expression of C-terminal-3HAtagged protein under its native promoter [73]. Transfected cells were selected under an increasing concentration of paromomycin (100 µg–1000 µg/ml) and decreasing concentration of CdCl_2_.

Living cells of *T. thermophila* were stained by Mitotracker Red CMXRos (Molecular Probes, Invitrogen) following the manufacturer’s protocol. Then, the cells were spread on polylysine-coated slides and immediately fixed using methanol, permeabilized with acetone, and immunostained by a α-HA tag rat monoclonal antibody (Roche) andAlexa Fluor 488 (green) donkey α-rat antibody (Invitrogen). Nuclei were stained with 4′,6-diamidin-2-fenylindol (DAPI). The slides were examined using an Olympus IX81 microscope equipped with an MT20 illumination system.

### *Phaeodactylum tricornutum* and *Thalassiosira pseudonana* cultivation, genetic manipulation, and microscopy

*Phaeodactylum tricornutum* (Bohlin, University of Texas Culture Collection, strain 646) and *Thalassiosira pseudonana* Hasle et Heimdal CCMP1335 were axenically grown in artificial seawater medium, made by dissolving “Tropic marine” salt (Wartenberg, Germany) to obtain 35 units of practical salinity and enriched by Guillard’s (F/2) Marine Water Enrichment Solution. The cells were cultivated at 22 °C under continuous illumination (80 mmol photons per m^2^ per s) with agitation (150 rpm) in 250 mL Erlenmeyer flasks to a density of approximately 7 × 10^6^ cells/ml.

*P. tricornutum* genes for Nbp35 (XP_002179311), mHCF101 (Joint Genome Institute, JGI portal ID 49,356), and chHCF101 (JGI portal ID 1865) and *T. pseudonana* genes for Nbp35 (XP_002289427), mHCF101 (XP_002290238), and chHCF101 (XP_002293925) were amplified from corresponding cDNA and cloned for expression in *P. tricornutum* with C terminal e-GFP in vector pPHA-NR4 [25]. Biolistic transfection was carried out as described previously [74] using M10 tungsten particles and 1350 psi rupture discs together with the Bio-Rad Biolistic PDS-1000/He particle delivery system. Transfected cells were grown at 22 °C under continuous illumination (80 mmol photons per m^2^ per s) on plates containing solid f/2-medium with 1.3% agar, 1.5 mM NH_4_^+^ as the sole nitrogen source and 75 µg/ml Zeocin™ as a selection marker. Protein expression under the control of the nitrate reductase promoter (pPha-NR vector) was induced by cultivation on 0.9 mM NO_3_ for 2 days.

Transformants were analyzed with a Leica TCSSP2 confocal laser scanning microscope. Mitochondrial localization was verified with MitoTracker® Orange CMTMRos (Life Technologies). The fluorescence of enhanced green fluorescent protein (eGFP) and chlorophyll was excited with an argon laser (65 mW) at 488 nm and detected with two photomultiplier tubes at bandwidths of 500 to 520 nm and 625 to 720 nm for eGFP and chlorophyll fluorescence, respectively. MitoTracker® Orange CMTMRos was excited with a HeNe (1.2 mW) laser at 543 nm, and emission was detected at 560–590 nm. Pictures were assembled in ImageJ (http://imagej.nih.gov/ij/index.html) using the Loci Bio-Formats plug-in (http://www.openmicroscopy.org/site/products/bio-formats).

### Searches for protein sequences and targeting predictions

Homologs of Ind1, Nbp35, Cfd1, and Hcf101 proteins were retrieved using the BLAST algorithm [75] from the National Center for Biotechnology Information (NCBI) nr database (https://www.ncbi.nlm.nih.gov/), JGI genome (https://genome.jgi.doe.gov/portal/), iMicrobe (https://www.imicrobe.us/), VEuPathDB (https://veupathdb.org/veupathdb/app) and Uniprot (https://www.uniprot.org/). Genes for *Cyanophora paradoxa* were obtained from the database at http://cyanophora.rutgers.edu/cyanophora/home.php. For each retrieved protein sequence, a given database, dataset, and gene number is indicated in Additional file 1: Table S1.

Protein sequences of four Nbp35-like protein categories were aligned using the multiple sequence comparison by log-expectation (MUSCLE) algorithm [76] in Geneious® 11.1.5 software with default settings. Protein sequences with incomplete N- terminal parts were excluded from further protein localization analysis. In a minority of cases, when N-terminal methionine was absent, but we identified methionine within the first 10 amino acids of the N-terminus, we shortened the sequence, and localization prediction was carried with lower confidence as indicated in Table 1. Subcellular targeting of proteins was predicted using TargetP-1.1 ([77], http://www.cbs.dtu.dk/services/TargetP-1.1/index.php); TargetP- 2.0 ([78],http://www.cbs.dtu.dk/services/TargetP/); DeepLoc-1.0 ([79],http://www.cbs.dtu.dk/services/DeepLoc/, accurate Profiles protein model); MitoFates ([80], http://mitf.cbrc.jp/MitoFates/cgi-bin/top.cgi); MitoProt ([81], https://ihg.gsf.de/ihg/mitoprot.html); SignalP 4.1 ([82], http://www.cbs.dtu.dk/services/SignalP-4.1/); SignalP 5 ([83], http://www.cbs.dtu.dk/services/SignalP/); Phobius ([84], http://phobius.sbc.su.se/); PSORT II ([85], https://psort.hgc.jp/form2.html); ChloroP ([86], http://www.cbs.dtu.dk/services/ChloroP/); Hectarv1.3 ([87], https://webtools.sb-roscoff.fr/); Multiloc ([88], https://omictools.com/multiloc-tool); and PlasmoAP ([89]; https://plasmodb.org/plasmo/plasmoap.jsp). Furthermore, proteins with detected signal peptides were shortened according to the predicted cleavage site of signal peptidase (SignalP 5, HECTAR, and TargetP 2 programs), and the presence of subsequent putative transit peptide was detected with the MitoFates, TargetP2, and ChloroP algorithms. A search for the motif of transit peptide cleavage by stromal processing peptidase was carried as described [90–92].

#### Phylogenetic analysis

For the initial analysis of Nbp-35-like proteins (Fig. 4), homologs of the ParA domain (PF10609) from the Pfam database were searched in the Uniprot database using HMMER (version 3). A total of 22328 sequences with e-values below the 1e-50 cutoff were selected. Selected sequences were grouped into groups that share 90% sequence identity using CD-HIT, and for each such group, one sequence was selected to reduce redundancy, resulting in a dataset of 9139 sequences. Sequences were then aligned using multiple alignment using fast Fourier transform (MAFFT) [93] with default settings, and the multiple sequence alignment was trimmed using block mapping and gathering with entropy (BMGE) [94] with the blocks substitution matrix (BLOSUM) 30 and a block size of one, resulting in an alignment with 189 aligned amino acid positions. Sequences that were aligned at less than 126 positions (more than 63 gaps) were removed from the dataset, resulting in 8440 sequences. These were again realigned and trimmed resulting in an alignment with 185 aligned amino acid positions. A phylogenetic tree was then inferred using FastTree [95] with default settings.

For detailed HCF101 analysis (Fig. 5), a dataset of 107 HCF101 proteins and their homologs was manually assembled. The sequences were aligned using MAFFT [93] with “–maxiterate 1000” and “–local pair” parameters. The alignment was trimmed using BMGE [94] with the BLOSUM30 matrix and a block size of one, which resulted in 311 aligned amino acid positions. A maximum likelihood phylogenetic tree was inferred using IQ-Tree (version 1.6) [96] with the best selected mixture model LG + C60 + G, and the topology was tested using 10 000 ultrafast bootstraps. A Bayesian phylogenetic tree was inferred using PhyloBayes (ver. 3) [97] and the CAT-Poisson model, running two chains for 20 000 generations. The first 2000 generations were discarded (burnin), and every tenth generation was sampled. The chains converged, with the maxdiff value 0.076.

#### Domain searches

Conserved protein domains were detected by searching sequences against the Pfam database (ver. 32) using HMMER (ver. 3) [98]. Hits with e-values below 1e−5 were considered.

## Supplementary Information

**Additional file 1: Table S1.** List of retrieved Nbp35-like protein sequences that were used for cellular localization predictions.

**Additional file 2: Table S2.** Predictions of domain structure in Nbp35 homologs included in phylogenetic analysis (Fig. 4).

## Data Availability

All data generated or analysed during this study are included in this published article and its supplementary information files.

## Abbreviations

BLOSUM: Blocks Substitution Matrix
BMGE: Block mapping and gathering with entropy
cDNA: Complementary DNA
Cpn60: Chaperonin 60
Cfd1: Cytosolic FeS cluster deficient 1
CIA: Cytosolic FeS cluster assembly
chHCF101: Chloroplastidial HCF101
DAPI: 4′,6-Diamidin-2-fenylindol
DUF: Domain of unknown function
EDTA: Ethylenediamine tetraacetic acid
eGFP: Enhanced green fluorescent protein
EGT: Endosymbiotic gene transfer
ERAD: Endoplasmic-reticulum-associated protein degradation
F1-ATPase: F1-adenosinetriphosphatase
FeS: Iron-sulfur
FSCA: FeS cluster assembly domain
HA: Hemagglutinin
HCF101: High chlorophyll fluorescence 101
ISC: FeS cluster assembly machinery
JGI: Joint Genome Institute
SAR: Stramenopila, Alveolata, Rhizaria
SUF: Sulfur utilization factor
LECA: Last eukaryotic common ancestor
LGT: Lateral gene transfer
MAFFT: Multiple alignment using fast Fourier transform
mHCF101: Mitochondrial HCF101
Mrp: MetG-related protein
MUSCLE: Multiple sequence comparison by log-expectation
NCBI: National Center for Biotechnology Information
Nbp35: Nucleotide-binding protein 35
NIF: Nitrogen-fixing
NTPases: Nucleoside-triphosphatase
PVC: Planctomycetes, Verrucomicrobia, Chlamydiae
SELMA: Symbiont-derived ERAD-like machinery
